# Histological-pathological and clinical T stage of primary adenoid cystic carcinoma of the lacrimal gland in a Chinese population

**DOI:** 10.1186/s12885-025-15426-9

**Published:** 2025-12-12

**Authors:** Jiayi Wu, Hui Cui, Meiqin Liang, Feng Wang

**Affiliations:** 1 Department of Ophthalmology, Changzhi People’s Hospital Affiliated to Changzhi Medical College, Changzhi, China; 2 Changzhi Medical College, Changzhi, China

**Keywords:** Lacrimal gland, Adenoid cystic carcinoma, Surgery, Pathology

## Abstract

**Background:**

To present clinical presentations, histological-pathological patterns, clinical T stage, divergent treating methods, and outcomes of primary adenoid cystic carcinoma (ACC) of the lacrimal gland in a Chinese population.

**Methods:**

This case series included patients with primary lacrimal gland adenoid cystic carcinoma treated at a Chinese hospital between 2003 and 2014. An exploratory multivariate Cox regression analysis was performed to evaluate the prognostic impact of clinical T-stage. Subsequently, Kaplan–Meier survival analysis was conducted, stratifying patients by T-stage (T4 vs. T1–T3) and by surgical approach among T4 patients, to assess the influence of tumor stage and surgical management on disease-free survival outcomes.

**Results:**

A total of 38 patients included 16 men and 22 women with a median age of 46.3 years were enrolled. Sixteen patients (42.11%) had local recurrence, while nineteen patients (50%) had distant metastasis at the time of presentation. Twelve patients (31.58%) were in T1-T3 stage and twenty-six (68.42%) were in T4 stage. Nineteen patients (73.08%) in the T4 stage exhibited a predominantly solid-basaloid pattern, and only three (25%) in T1-T3 stage had a predominantly solid-basaloid pattern histological pattern. Median DFS for the entire cohort was 29.0 months (95% CI, 19.0–39.0), and median survival after metastasis was 7.0 months (95% CI, 3.0–9.0). After adjustment for covariates, T4 stage remained independently associated with significantly shorter DFS (HR = 4.46, 95% CI: 1.40-14.21, *P* = 0.011). A significant difference in DFS was observed between the T1–T3 and T4 groups (log-rank *P* = 0.003). Meanwhile, no significant difference in disease-free survival was observed between T4 patients undergoing globe-preserving surgery and eye-sparing approaches (log-rank *P* = 0.297).

**Conclusions:**

In this Chinese cohort, the solid-basaloid pattern correlated strongly with advanced T4 disease and aggressive behavior. Kaplan–Meier and multivariate Cox analyses consistently demonstrated that T4 stage was independently associated with significantly poorer DFS. For T4 patients, DFS did not differ significantly between exenteration and globe-preserving surgery when combined with radiotherapy, suggesting that eye-sparing approaches may be viable in advanced cases.

**Supplementary Information:**

The online version contains supplementary material available at 10.1186/s12885-025-15426-9.

## Introduction

Adenoid cystic carcinoma (ACC) is a rare form of adenocarcinoma and is the most common malignant epithelial tumor of the lacrimal gland which represents about 1.6% of all orbital tumors and 29% of all epithelial tumors of the lacrimal gland [1]. The tumor generally presents in either a solid pattern or cribiform pattern (more holey) with survival being longer in those with the cribiform pattern [2]. Overall mortality associated with lacrimal ACC tumors are reported up to 87% despite maximum treatment [3–5]. Lacrimal adenoid cystic carcinoma is an aggressive, slow-growing and painless tumor with a potential to invade into the surrounding intracranial tissues often with grave outcomes [6, 7]. Patients may present with nebulous symptoms including asymmetric orbital pain, double vision, ptosis, and decreased visual acuity [8]. Because of its rarity and non-specific presentation, a diagnosis is often difficult and frequently initially incorrect which can lead to poorer outcomes [9, 10]. Studies of lacrimal ACC often include a very limited number of patients [11]. Clinically diagnosing lacrimal ACC is done via the seventh edition of the American Joint Committee on Cancer (AJCC) Staging Manual which defines T stage based upon tumor size and its potential extensions [12, 13].

While controversy exists over the appropriate management for adenoid cystic carcinoma of the lacrimal gland, as existing evidence is predominantly derived from Western populations [14], data on its presentation and behavior in Chinese patients remain scarce. Preliminary observations suggest that Chinese patients may more frequently present with advanced-stage disease, although this has not been systematically investigated [15]. This study therefore aimed to characterize the clinical and histopathological features of ACC in a Chinese cohort, with a particular emphasis on the correlation between the solid-basaloid pattern and advanced clinical T-stage. Furthermore, we evaluated treatment outcomes between globe-preserving surgery and exenteration in a patient population predominantly presenting with advanced (T4) disease, a distinctive feature of our cohort.

## Subjects and methods

### Study design and patients

All procedures were performed in accordance with the ethical standards laid down in the 1964 Declaration of Helsinki and its later amendments. This case series study reviewed the medical records of patients with primary ACC of the lacrimal gland who were treated in Changzhi People’s Hospital in China between 2003 and 2014. A total of 48 patients with lacrimal gland adenoid cystic carcinoma were initially identified; 6 were excluded due to incomplete follow-up information and 4 were excluded due to unavailable pathological slides, leaving 38 patients for the final analysis. Prior to study onset, approval for the study was obtained from the Ethics Committee-Institutional Review Board (HEC-IRB) of the respective hospital. The reporting of this study follows the STROBE guidelines for observational studies.

### Data collection

The pathology slides were examined by one of us, without prior knowledge of the clinical patient data. Clinical variables collected from medical records included age, gender, size of the lesion, presenting symptoms (pain, proptosis, diplopia, decreased visual acuity), histological characteristics, methods of treatment, the presence of perineural invasion, the presence of adequate free margin in specimen, occurrence of local, regional, or distant metastasis, disease-free and overall survival (OS) time. Disease-free survival (DFS) was defined as time from the completion of treatment, and OS was calculated from date of initial diagnosis. Histopathological classification was performed according to the WHO criteria. The solid subtype, also known as the basaloid pattern, is characterized by nests and sheets of small, hyperchromatic cells with scant cytoplasm, often with central necrosis [16]. The term ‘solid-basaloid’ is used throughout this article to denote this pattern. Tumor staging was based upon the AJCC classification system *(AJCC Cancer Staging Manual*, 7th edition) (Table S1) [17].

#### Statistical analysis

Continuous variables, including age, DFS, and post-metastatic survival time, were summarized as median values, whereas follow-up duration and recurrence intervals were expressed as mean ± standard deviation (SD). Categorical variables were compared using the Chi-square test or Fisher’s exact test, and continuous variables were analyzed using the independent-samples t-test. To account for potential confounding factors, an exploratory multivariate Cox proportional hazards regression model was additionally constructed. The model incorporated age, sex, perineural invasion (PNI), bone invasion, and margin status as covariates to further assess the independent effect of clinical T stage on DFS. Kaplan–Meier analysis was performed to estimate both OS and DFS of 38 patients. The log-rank test was used to compare survival distributions. Numbers at risk and event counts were displayed beneath each Kaplan–Meier curve. All confidence intervals were estimated using the log-log transformation method. In addition to overall analyses, Kaplan–Meier survival analysis was conducted, stratifying patients by T-stage (T4 vs. T1–T3) and by surgical approach among T4 patients, to assess the influence of tumor stage and surgical management on DFS. Statistical analyses were performed using SPSS version 26.0 and R version 4.5.1. A two-tailed *P*<0.05 was considered statistically significant.

## Results

**Fig. 1:**
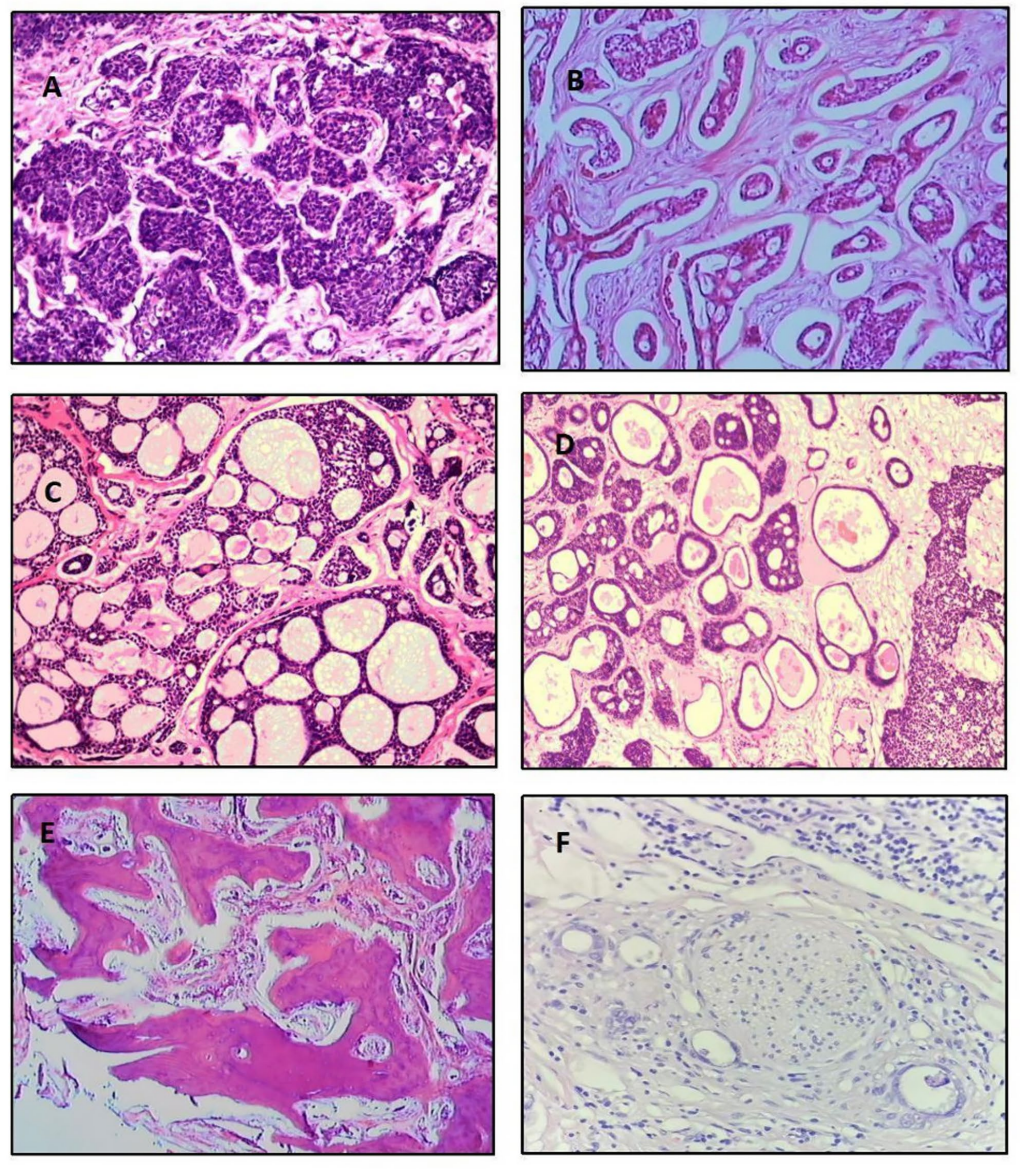
Representative images of adenoid cystic carcinoma with HE staining. **A**: solid-basaloid pattern; **B**: tubular pattern;**C**: cribriform pattern; **D**: combination of tubular pattern and cribriform pattern;**E**: Orbital bone invaded by tumor cells; **F**: Perineural sheath of orbital nerves invaded by tumor cells. Scale bars: 50µm (**A-C**), 100µm (**D-F**); Original magnification: 100× (**A-C**), 50× (**D**), 40× (**E-F**)

This study included 22 females and 16 males with a median age of patients at diagnosis of 46.3 years (14 ~ 73 years). None of 38 patients had bilateral involvement. 18 patients had left eye tumor involvement and 20 were right eye. The most common presenting symptoms was proptosis (78.94%), followed by pain (68.42%), diplopia (36.84%), and decreased visual acuity (34.21%) (Table 1). At histopathological examination 22 patients (57.89%) presented with a solid-basaloid pattern (Fig. 1A; Table 1), 5 (13.16%) with a tubular pattern (Fig. 1B; Table 1), 2 (5.26%) had a cribriform pattern (Fig. 1C; Table 1), and 9 (23.68%) a mixed pattern (Fig. 1D; Table 1), respectively. Furthermore, nineteen patients (76%) in T4 stage had a predominantly solid-basaloid pattern (Table 1). The cribriform and tubular patterns tended to be skewed more frequently to the lower stage Bone invasion (Fig. 1E) was clinically or pathologically apparent in 81% (30/38) of the tumors, and nerve invasion (Fig. 1F) was seen in 52.6% (20/38) of the tumors.

**Table 1:** Patient demographics and outcomes

Characteristics and outcomes	All patients (*n* = 38)
Age ^a^	46.3 (14–73)
Sex ^c^	
Female	22 (57.89%)
Male	16 (42.11%)
Affected side ^c^	
Right	20 (52.63%)
Left	18 (47.37%)
Presenting symptoms ^c^	
Proptosis	30 (78.94%)
Pain	26 (68.42%)
Diplopia	14 (36.84%)
Decreased visual acuity	13 (34.21%)
Clinical T stage ^c^	
T1	3 (7.89%)
T2	4 (10.52%)
T3	5 (13.16%)
T4	26 (68.42%)
Histologic subtype ^c^	
Cribriform	2 (5.26%)
solid-basaloid	22 (57.89%)
Tubular	5 (13.16%)
Mixed	9 (23.68%)
Treatment ^c^	
R + RT	24 (63.15%)
E + RT	14 (36.85%)
Time (mo) of follow-up ^b^	44.1 ± 26.2
Time (mo) of recurrence ^b^	21.1 ± 11.0
Local recurrence ^c^	
Yes	16 (42.11%)
No	22 (57.89%)
5-year survival rate ^c^	
Yes	20 (52.63%)
No	18 (47.37%)
Median disease-free survival time, total (mo) ^d^	29.0 (19.0–39.0)
Patients in T1 ~ T3 stage ^d^	44.0 (21.0–75.0)
Patients in T4 stage ^d^	21.0 (16.0–32.0)
Location of distant metastasis ^c^	
Lung	10 (26.32%)
Liver	5 (13.16%)
Bone	4 (10.53%)
Kidney	3 (7.89%)
Brain	2 (5.26%)
Lymph nodes	2 (5.26%)
Total	19 (50%)
Median survival time after metastases (mo) ^d^	7.0 (3.0–9.0)

R + RT, globe-preserving apparent gross tumor resection with postoperative radiation therapy; E + RT, exenteration with removal of the bone of the superior and lateral orbit and postoperative radiation therapy

^a^ Continuous variables are described using the mean (minimum, maximum)

^b^ Continuous variables are described using the mean (± standard deviation)

^c^ Categorical variables are described using n (%)

^d^ Continuous variables are described using the median (95% CI)

**Fig. 2:**
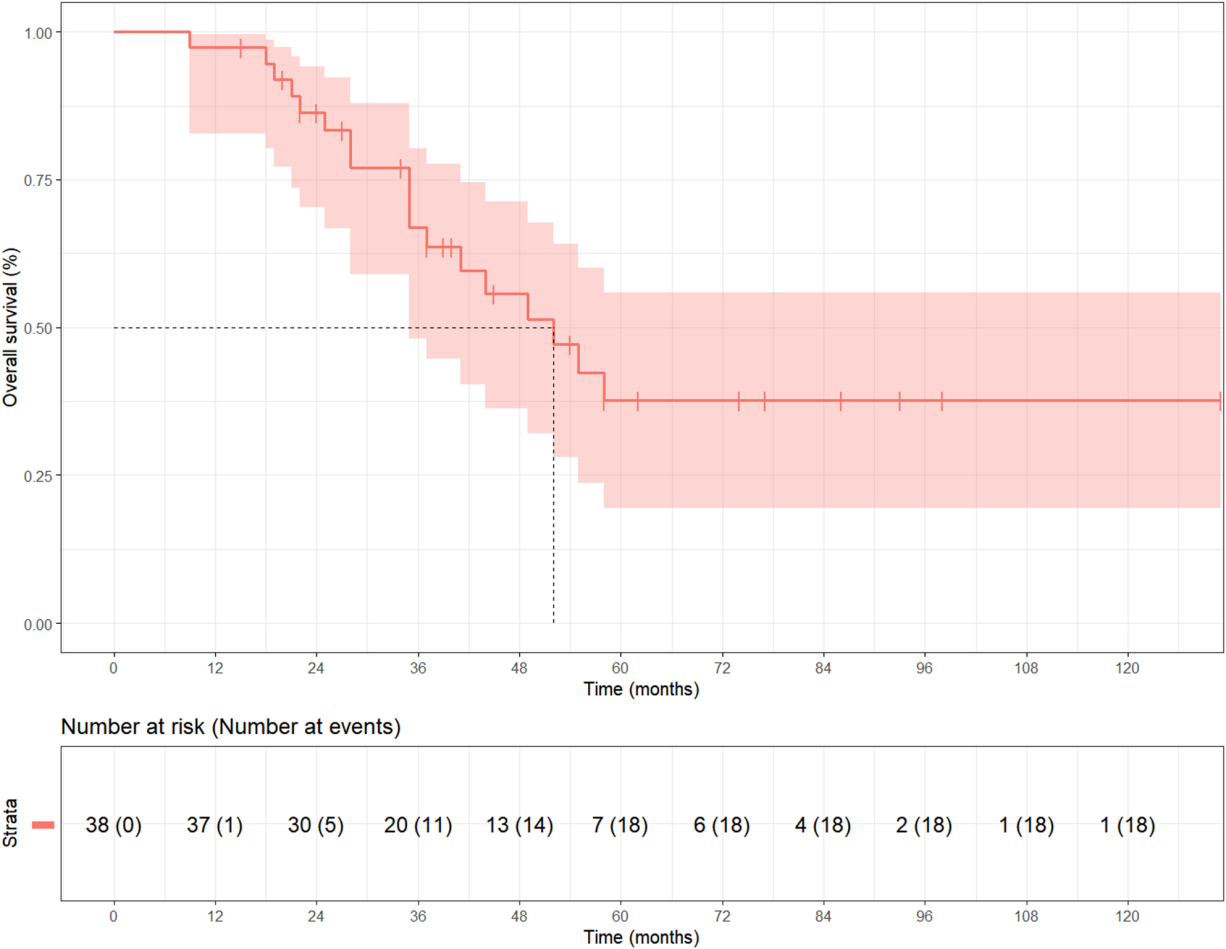
Kaplan-Meier test showing overall survival of all 38 patients

Sixteen patients (42.11%) developed regional recurrence and nineteen patients (50%) had distant metastasis. Common sites of metastasis included lung (26.32%), followed by liver (13.16%), bone (10.53%), kidney (7.89%), brain (5.26%), and lymph nodes (5.26%). Among the 20 T4-stage patients who experienced local recurrence or distant metastasis, 18 exhibited a solid-basaloid pattern. The median survival after diagnosis of metastatic disease was 7.0 months (95% CI, 3.0–9.0). The median DFS time for the entire group was 29.0 months (95% CI, 19.0–39.0). The 5-year survival rate in this series was 52.63% (Table 1; Fig. 2).

**Fig. 3:**
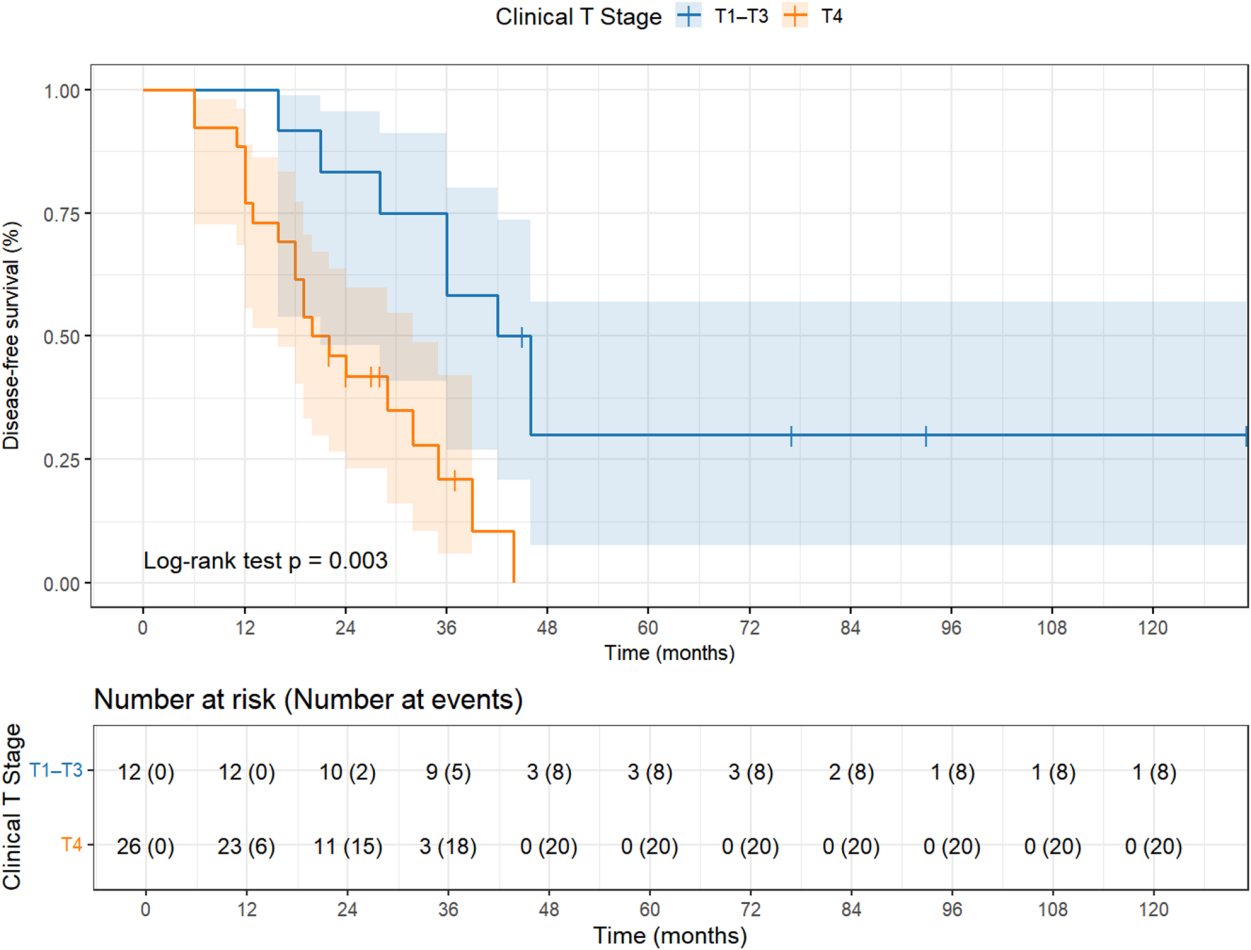
Kaplan–Meier survival curves showing disease-free survival of all 38 patients stratified by T stage (T4 vs. T1-T3)

This study illustrates OS of all 38 patients, whereas Fig. 3 depicts the stratified DFS according to T stage (T4 vs. T1-T3) with the number at risk displayed below each curve. At the time of presentation, 3 cases (7.89%) were in T1 stage, 4 cases (10.53%) were in T2 stage, 5 cases (13.16%) were in T3 stage and 26 cases (68.42%) were in T4 stage. Kaplan–Meier survival curves stratified by T stage (T4 vs. T1-T3) demonstrated a significant difference in DFS between the two groups (log-rank *P* = 0.003; Fig. 3). The median DFS for patients in T4 stage was 21.0 months (95% CI, 16.0–32.0) and for patients in T1–T3 stage was 44.0 months (95% CI, 21.0–75.0). To further evaluate the independent prognostic value of T stage, an exploratory multivariate Cox proportional hazards analysis was performed. After adjusting for age, sex, perineural invasion, bone invasion, and margin status, T4 stage remained independently associated with significantly worse DFS (HR = 4.46, 95% CI: 1.40–14.21, *P* = 0.011). Perineural invasion also showed an independent effect on DFS (HR = 2.78, 95% CI: 1.07–7.24, *P* = 0.036). None of the other covariates reached statistical significance (Table 2).

**Table 2:** Multivariable Cox proportional hazards analysis for disease-free survival (*n* = 38)

Variable	β (SD)	HR (95% CI)	*P* value
Age (per year increase)	−0.02 (0.01)	0.98 (0.96–1.01)	0.183
Sex (Male vs. Female)	0.38 (0.46)	1.46 (0.59–3.59)	0.413
Perineural invasion (Yes vs. No)	1.02 (0.49)	2.78 (1.07–7.24)	0.036
Bone invasion (Yes vs. No)	−0.28 (0.56)	0.76 (0.25–2.27)	0.620
Positive resection margin (Yes vs. No)	−0.32 (0.45)	0.72 (0.30–1.75)	0.473
Clinical T stage (T4 vs. T1–T3)	1.50 (0.59)	4.46 (1.40–14.21.40.21)	0.011

This multivariable cox regression model was attempted to adjust for known prognostic factors,including age, sex, clinical T stage, positive resection margin, perineural invasion, and boneinvasion. HR: Hazard ratio; 95% CI: 95% Confidence Interval

**Fig. 4:**
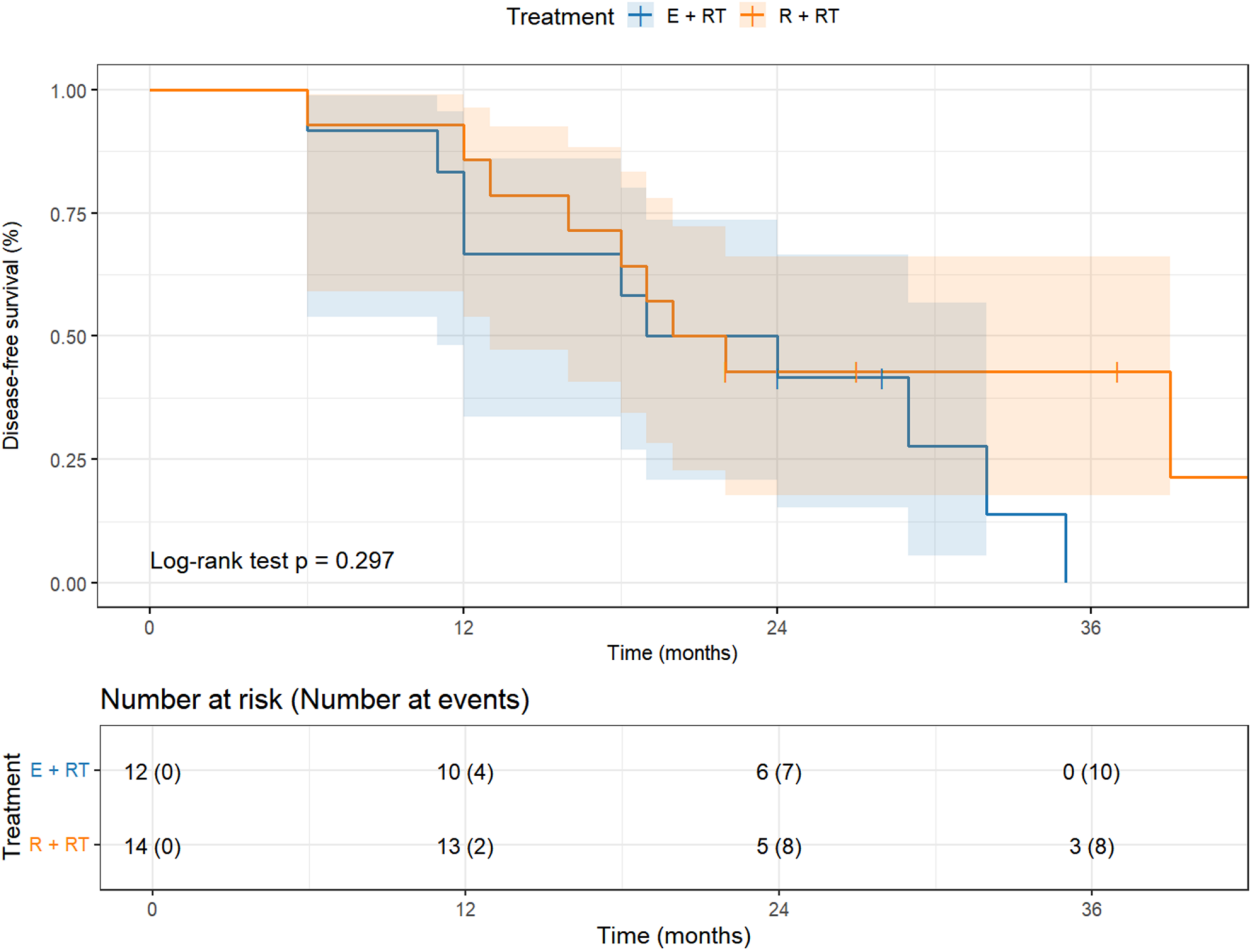
Kaplan–Meier survival curves showing disease-free survival of T4 patients stratified by Treatment

Moreover, the median DFS for patients in T4 who had undergone globe-preserving apparent gross tumor resection followed by postoperative radiation therapy were 21.5 months (95% CI, 11.0–32.0), and for patients who underwent exenteration with bone removal were 21.0 months (95% CI, 13.0–30.0) which was not significantly (*P* = 0.297) (Fig. 4).

## Discussion

This study characterizes lacrimal gland adenoid cystic carcinoma in a homogeneous Chinese cohort, marked by a high frequency of advanced T4 disease at diagnosis. Key findings include a strong association between the solid-basaloid histologic pattern and T4 stage, observed in 76% (19/25) of T4 cases. Moreover, among T4 patients, the extent of surgery (exenteration vs. globe-preserving resection) did not significantly influence DFS when combined with adjuvant radiotherapy. Multivariate analysis confirmed T4 stage as an independent predictor of poorer DFS, after adjusting for clinicopathological variables. Consistently, Kaplan–Meier analysis revealed significantly worse survival in T4 versus T1–T3 patients, underscoring the prognostic importance of T classification. However, within this high-risk T4 subgroup, the extent of surgery (exenteration versus globe-preserving resection) did not confer a significant DFS advantage when combined with adjuvant radiotherapy.

Although the association between the solid-basaloid pattern and aggressive behavior is well-documented [18], our study provides cohort-specific evidence by demonstrating its overwhelming prevalence (19/26, 76%) in patients presenting with advanced T4 disease. This underscores its role not just as a marker of biological aggressiveness, but also as a potential indicator of advanced clinical stage at diagnosis in our population. Adenoid cystic carcinoma is the most common malignant tumor of the lacrimal gland and overall, the prognosis for this pathology very poor [6]. Lacrimal ACC’s peak incidence occurs in the fourth decade of life [19] with the mean age at diagnosis in this case series (46.3 years) being similar to most other reported series in adults. Some literature has suggested that the disease is more common in women [20] which comports with the ratio of female patients (57.89%) who had ACC tumors in the present study. Wright et al. [21] emphasized that pain is a frequently reported symptom of lacrimal gland malignancies given its perineural infiltration. In the current study, the most common presenting symptoms was proptosis (83.33%), followed by pain (52.63%) which is consistent with rates reported by Wright et al. In light of the poor prognosis of lacrimal gland ACC, it is suggested that a 40-year-old female patient presenting with symptoms of proptosis with a painful palpable mass in supratemporal quadrant of the orbit be regarded as having a malignant neoplasm until proven otherwise. Perineural invasion also indicated higher risk of recurrence and mortality. Ahmad et al. [22] report that 93% of local recurrence had histological evidence of perineural invasion and 53% of local recurrence had failure to obtain free margin. In our study, 87.50% (14/16) of local recurrence had histological evidence of perineural invasion. Solid-basaloid pattern predominant subtype was associated with histological tumor-cell-free margin and absence of perineural invasion. As shown in the study, the mortality rate in patients without perineural invasion was 33.33% (6/18), which is lower than the rate observed among patients with perineural invasion (12/20, 60%). However, due to the limited sample size and varying disease severity, the findings of this study require further validation in multi-center studies.

The histopathological spectrum of lacrimal gland ACC classically encompasses three subtypes: cribriform, tubular, and solid-basaloid. While the cribriform pattern is frequently reported as the most common subtype in the literature [23], the solid-basaloid pattern predominated in our cohort (57.89%, 22/38). This discrepancy may be attributed to the unique characteristic of our study population, namely that a substantial majority (68.42%, 26/38) presented with advanced (T4) disease—a clinical setting where this more aggressive variant is frequently observed. Indeed, our data strongly support this association, as 76% (19/26) of T4 patients exhibited a predominantly solid-basaloid pattern. We speculate that the aggressive behavior of the solid-basaloid pattern, characterized by its tendency for extraluminal growth and invasion, underlies its strong correlation with advanced T4 staging at presentation and the observed shorter DFS in these patients. The multivariate Cox analysis further substantiates this link, demonstrating that T4 stage independently predicts a significantly shorter DFS, even when histopathological parameters such as PNI, bone invasion, and margin status are accounted for. Although the AJCC Staging Manual (7th edition) extends beyond purely anatomical criteria [17], our findings highlight the critical importance of integrating histopathological pattern—particularly the identification of the solid-basaloid variant—into clinical staging and prognostic assessment, as it may signify a more aggressive disease course.

Management of adenoid cystic carcinoma of the lacrimal gland remains controversial [24]. Some authorities advocate conservative surgical therapy followed by external-beam radiation therapy. Others believe that radical surgery may result in better long-term survival [25]. In this small retrospective cohort, we did not observe a significant difference in survival between radical surgery and conservative surgery. The median DFS for T4 patients who underwent globe-preserving apparent gross tumor resection was 21.5 months (95% CI, 11.0–32.0), and patients who underwent exenteration were 21.0 months (95% CI, 13.0–30.0). Thus, it seems unlikely that radical surgical therapy would have a beneficial effect on long-term survival. The type of radical surgery required in the treatment of ACC of the lacrimal gland often evokes negative emotions, especially in female patients may regard as experience. We posit that one of the effects of these intense negative reactions and moods was to shorten long-term survival amongst those patients. Adjuvant radiotherapy is recommended after surgical resection, regardless of its extent, especially for patients at high risk of recurrence [25]– [26]. Adjuvant radiation therapy has proven to be effective in preventing locoregional recurrences with local control rates of approximately 50%–80% at 5 years, despite no benefit in OS [5].

The major challenge in the treatment of adenoid cystic carcinoma of the lacrimal gland is prevention of systemic metastasis. Despite aggressive local treatment, adenoid cystic carcinoma of the lacrimal gland spawn a high rate of local recurrence and late distant metastasis, with significant resultant mortality [27]. Lung is the most common site of metastasis, with the liver being the second most common site. Of the patients reviewed for this study, only 2 had lymph node and brain metastasis. Some investigators have advocated the use of neutron beam therapy as a possibly more effective form of delivery of radiation therapy. All patients received postoperative radiation therapy in this study. The 5-year survival rates in this case series were 52.63%, which consistent with rates reported by Esmaeli et al. [10] who reported that the median DFS was 18 months. While this case series showed higher median survival for patients than reported previously, and the median survival after diagnosis of metastatic disease lower.

Several limitations of this study should be acknowledged. First, the small sample size (*n* = 38) limited the statistical power for subgroup analyses, and the limited number of outcome events may have reduced the reliability of the multivariate Cox model, which should therefore be considered exploratory. Second, as a single-center retrospective study, the findings may be subject to institutional and regional biases, necessitating validation through future multi-center studies. Third, detailed radiotherapy parameters were not systematically documented, preventing their inclusion in the multivariate analysis. Finally, the extended study period (2003–2014) and retrospective design introduced inherent constraints: the median follow-up was insufficient to capture very long-term outcomes beyond five years, and it was not feasible to account for evolving surgical and radiotherapy techniques over time.

In this Chinese cohort, primary lacrimal gland ACC exhibited a pronounced association between the solid-basaloid histological pattern and advanced T4 disease at presentation, underscoring its role as a marker of biological aggressiveness. Multivariate Cox analysis and Kaplan-Meier curves consistently identified T4 stage as an independent predictor of shorter DFS, underscoring its prognostic primacy. However, within this high-risk T4 subgroup, the extent of surgery (exenteration versus globe-preserving resection) showed no significant impact on DFS when combined with adjuvant radiotherapy. These findings emphasize the prognostic value of histopathological subtyping and suggest that less radical, eye-sparing surgery may be a viable option for advanced-stage disease without compromising oncologic outcomes in the short term. However, its ultimate clinical adoption should be considered in conjunction with surgeon expertise, patient preference, and prospective evidence on functional outcomes.

## Supplementary Information

Supplementary Material 1.

## Data Availability

All original data generated or analyzed during the current study are available from the corresponding author upon reasonable request.
