# Supplementary material for: Histological-pathological and clinical T stage of primary adenoid cystic carcinoma of the lacrimal gland in a Chinese population
Source: BMC Cancer. 2025 Dec 12;26:110. doi: 10.1186/s12885-025-15426-9 (PMC12831414; doi:10.1186/s12885-025-15426-9)

**Table S1.** T stage definitions for lacrimal gland tumors

| Primary tumor (T) | |
| --- | --- |
| TX | Primary tumor cannot be assessed |
| T0 | No evidence of primary tumor |
| T1 | Tumor 2 cm or smaller in greatest dimension, with or without extraglandular extension into the orbital soft tissue |
| T2 | Tumor larger than 2 cm but not larger than 4 cm in greatest dimension |
| T3 | Tumor larger than 4 cm in greatest dimension |
| T4 | Tumor invades periosteum or orbital bone or adjacent structures |
| T4a | Tumor invades periosteum |
| T4b | Tumor invades orbital bone |
| T4c | Tumor invades adjacent structures (brain, sinus, pterygoidfossa, temporal fossa) |

**Table S2.** **Individual patient characteristics and clinical outcomes**

| Patient | Sex | Age range | Affected side | Clinical T stage | Histologic subtype | Treatment | Time (mo) of local recurrence | Time (mo) site of metastases | Time (mo) of follow-up | status |
| --- | --- | --- | --- | --- | --- | --- | --- | --- | --- | --- |
| 1 | F | 40s | R | T1 | Cribriform | R +RT | No | No | 131 | + |
| 2 | M | Early adolescence | L | T1 | Basaloid | R +RT | No | 36;Lung | 39 | + |
| 3 | F | 40s | R | T1 | Tubular | R +RT | No | No | 93 | + |
| 4 | M | 50s | L | T2 | Cribriform | R +RT | 46; LR | No | 58 | + |
| 5 | F | 30s | R | T2 | Tubular | R +RT | No | 42; Lung | 55 | － |
| 6 | F | Early adolescence | R | T2 | Tubular | R +RT | No | No | 45 | + |
| 7 | M | 20s | L | T2 | Basaloid | R +RT | 21; LR | No | 54 | + |
| 8 | M | 50s | R | T3 | Mixed | R +RT | 16; LR | No | 40 | + |
| 9 | F | 60s | L | T3 | Tubular | R +RT | No | 36; Lymph node | 49 | － |
| 10 | M | 50s | L | T3 | Mixed | E+RT | 28; LR | No | 98 | + |
| 11 | M | Late adolescence | L | T3 | Tubular | R +RT | No | No | 77 | + |
| 12 | F | 50s | R | T3 | Basaloid | E+RT | No | 46; bone, kidney | 52 | － |
| 13 | F | 50s | R | T4 | Basaloid | E+RT | 12; LR | No | 15 | + |
| 14 | M | 60s | R | T4 | Basaloid | R +RT | No | No | 22 | + |
| 15 | F | 60s | L | T4 | Basaloid | E+RT | No | 6; kidney | 18 | － |
| 16 | F | 20s | R | T4 | Basaloid | E+RT | 11; LR | 16; bone | 19 | － |
| 17 | F | 20s | R | T4 | Mixed | R +RT | No | 20; liver | 28 | － |
| 18 | M | 50s | L | T4 | Basaloid | R +RT | 16; LR | No | 62 | + |
| 19 | M | 30s | R | T4 | Basaloid | E+RT | 18; LR | No | 20 | + |
| 20 | F | 30s | L | T4 | Basaloid | E+RT | No | 29;Lymph node | 37 | － |
| 21 | M | 20s | L | T4 | Basaloid | R +RT | 13; LR | No | 34 | + |
| 22 | F | 30s | L | T4 | Basaloid | E+RT | 12; LR | 28; Lung | 35 | － |
| 23 | F | 40s | R | T4 | Mixed | R +RT | No | 12;bone | 21 | － |
| 24 | F | 30s | L | T4 | Mixed | R +RT | No | No | 27 | + |
| 25 | M | 50s | R | T4 | Basaloid | R +RT | 18; LR | 29; Lung, liver | 35 | － |
| 26 | M | 30s | R | T4 | Basaloid | R +RT | No | No | 27 | + |
| 27 | F | 40s | L | T4 | Mixed | E+RT | 35; LR | 49; Lung, kidney | 58 | － |
| 28 | F | 60s | L | T4 | Basaloid | R +RT | No | 19; Lung | 22 | － |
| 29 | M | 50s | R | T4 | Basaloid | R +RT | No | No | 37 | + |
| 30 | F | 20s | L | T4 | Basaloid | E+RT | No | 19; liver | 25 | － |
| 31 | F | 30s | R | T4 | Mixed | E+RT | 24; LR | 69;Lung, bone | 74 | + |
| 32 | F | 60s | L | T4 | Basaloid | R +RT | 39; LR | No | 86 | + |
| 33 | M | 60s | R | T4 | Mixed | E+RT | No | 32; Lung, brain | 35 | － |
| 34 | M | 70s | R | T4 | Basaloid | E+RT | No | No | 24 | + |
| 35 | F | 60s | L | T4 | Basaloid | R +RT | 22; LR | 33; Lung, liver | 41 | － |
| 36 | F | 50s | R | T4 | Basaloid | R+RT | No | 6; brain | 9 | 0 |
| 37 | M | 70s | R | T4 | Mixed | E+RT | No | No | 28 | － |
| 38 | F | 60s | L | T4 | Basaloid | R+RT | 6; LR | 44; Lung, liver | 44 | 0 |

R + RT, globe-preserving apparent gross tumor resection + postoperative radiation therapy; E + RT, exenteration with removal of the bone of the superior and lateral orbit + postoperative radiation therapy; LR, local recurrence; +, Alive; －, Deceased because of disease.

**Figure S1.** Kaplan-Meier test showing disease-free survival of all 38 patients.


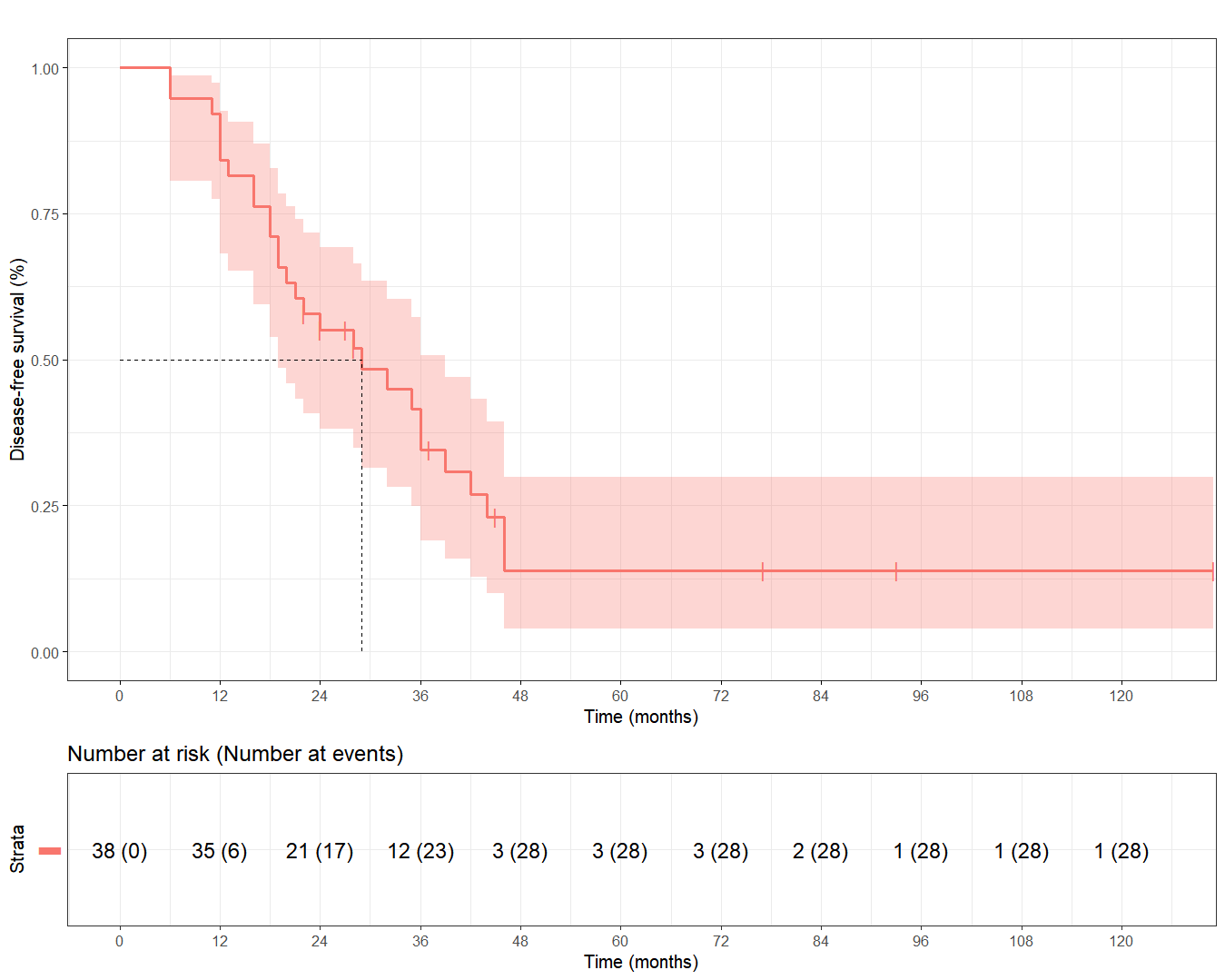

Supplement: Supplementary file 1 — Supplementary Material 1. [file 12885_2025_15426_MOESM1_ESM.docx]
